# Intrasurgical Seminiferous Tubular Diameter Correlates with Total Motile Sperm Count in Azoospermia: a Prospective Cohort Study

**DOI:** 10.1007/s43032-022-00927-w

**Published:** 2022-03-31

**Authors:** Medhat Amer, Sameh Fayek GamalEl Din, Ashraf Zeidan, Ahmed Adel, Islam Elsisi, Emad Fakhry, Ahmed Raef Sadek

**Affiliations:** 1 Andrology & STDs Department, KasrAlAiny Faculty Of Medicine, Cairo, Egypt; 2 Adam International Hospital, Giza, Egypt

**Keywords:** Micro-TESE, Non-obstructive azoospermia (NOA), Seminiferous tubular diameter, Sperm retrieval rate (SRR)

## Abstract

We aimed to find a correlation between the intraoperative diameters of the seminiferous tubules evaluated at high magnification during micro-testicular sperm extraction (micro-TESE) and total motile sperm count (TMSC) in non-obstructive azoospermic (NOA) patients. Five hundred four consecutive NOA patients were included and underwent micro-TESE. The change in the mean TMSC and different seminiferous tubular diameters was of high statistical significance (*p* < 0.001). It should be noted that the highest mean TMSC was reported in the dilated tubules (DTs) group followed by the other study groups 941.72 ± 196.97, 487.37 ± 443.57, and 34.54 ± 60.79, respectively. Furthermore, 21 naïve cases had dilated tubules (DTs) and 18 (85.7%) of them had shown eventful micro-TESE. Conversely, 186 naïve cases had slightly dilated tubules (SDTs), and 101 (54.3%) of them had eventful micro-TESE. Only 8 (24.25%) cases of the 33 cases had non dilated tubules (NDTs) and showed eventful micro-TESE. The frequency of intrasurgical seminiferous tubular diameter and micro-TESE outcome among the naïve cases had demonstrated a highly statistical significance (*p* < 0.001). Interestingly, all salvaged cases (100%) with DTs and a previous eventful TESE had shown eventful TESE in the current study. The most dilated intrasurgical seminiferous tubular diameter is associated with the highest TMSC in NOA patients including SCO cases.

## Introduction

Microdissection testicular sperm extraction (micro-TESE) is the gold standard surgical technique for non-obstructive azoospermia (NOA) patients with variable sperm retrieval rates (SRRs) among centers [1]. The probability of successful sperm retrieval (SSR) is 1.5 times using micro-TESE compared to conventional TESE [2]. This higher SSR is due to the optical magnification (15–24 ×) of the testicular parenchyma that helps the identification of dilated and sometimes opaque tubules, which are presumed to contain mature germ cells [3, 4]. Detection of the prevalent seminiferous tubule caliber pattern using high optical magnification has attracted the attention of few authors in the past decade due to it’s potential ability to explain the outcome of micro-TESE in NOA subjects [5–7]. Consistently, a previous study had shown that selecting and isolating the most dilated and opaque seminiferous tubules using the surgical loop coupled with laboratory stereoscopic dissection led to better sperm retrieval in NOA men [8]. We aimed in this prospective cohort study to find a correlation between the intrasurgical diameter of the seminiferous tubules evaluated at high magnification during micro-TESE and total motile sperms count (TMSC) in NOA patients either naïve or salvaged cases.

## Methods

A prospective cohort study was conducted where it included 504 consecutive patients attending Adam International Hospital. All participants were males complaining of primary infertility diagnosed as NOA. An informed consent was signed by all participants in the study which explained thoroughly the steps of the study and the possible beneficial outcomes expected which was approved by our institutional review board (I-071018) and conformed to Helsinki declaration (2013) [9].

### Inclusion Criteria

**Fig. 1:**
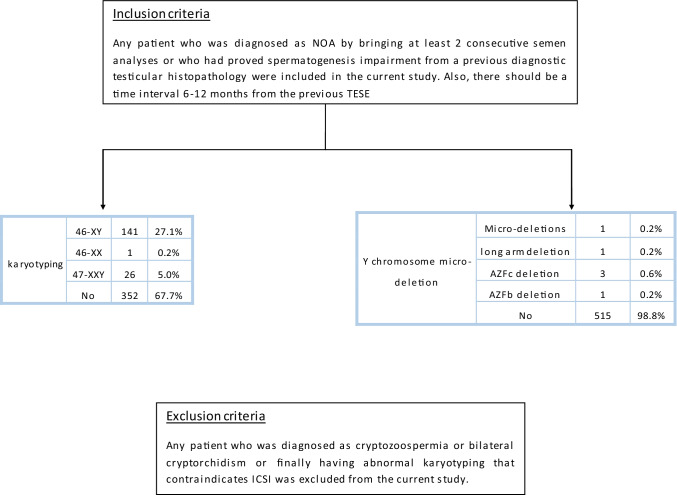
Flowchart of the inclusion and exclusion criteria of the study

Any patient who was diagnosed as NOA by bringing at least 2 consecutive semen analyses or who had proved spermatogenesis impairment from a previous diagnostic testicular histopathology were included in the current study. Also, there should be a time interval 6–12 months from the previous TESE (Fig. 1). [10, 11].

### Exclusion Criteria

Any patient who was diagnosed as cryptozoospermia or bilateral cryptorchidism or finally having abnormal karyotyping that contraindicates ICSI was excluded from the current study (Fig. 1).

All patients were subjected to a complete medical history and both general and local genital examinations to evaluate potentially correctable causes of infertility. Testicular volume was determined sonographically using Lambert formula = length × width × height × 0.71 as it is more sensitive than Prader orchidometery [12, 13]. Moreover, a testicular volume of ≥ 12.5 cm^3^ was considered normal and less than that as small [14]. Additionally, serum follicle stimulating hormone (FSH), luteinizing hormone(LH), prolactin, estradiol (E2), total testosterone, and free testosterone levels were evaluated using chemiluminescence immunoassay (CLIA) technique (1.5–14 mIU/ml for FSH, 1.5–8 mIU/ml for LH, 2.5–17 ng/ml for prolactin, 2.4–8.3 ng/ml for total testosterone, and 20–47 pg/ml for estradiol). A fasting morning serum sample for basal hormones determination was obtained prior to the micro-TESE attempt. All assays were performed using Cobas E411 immunoassay analyzer (Roche Diagnostics GmbH, Mannheim, Germany).

Moreover, Giemsa Karyotype was used for standard cytogenetic procedure in all cases by analysis of at least 20 G-banded metaphases from a peripheral blood lymphocyte culture, and in all cytogenetically normal cases, molecular screening for Yq microdeletions was carried out on DNA extracted from peripheral blood using PCR [15]. Micro-TESE was performed under general anaesthesia with the patient in a supine position. A floor-standing operating microscope (Leica M500; Leica microsystems Pty Ltd, Gladesville, NSW, Australia) was used throughout the procedures. A micro meter was placed on the eye pieceusing X24 (dual-headed binocular tube and eyepieces 200-, 300-, 350-mm objective lens, motorised foot-operated zoom system) to determine the diameter of the seminiferous tubules [7]. During micro-TESE, if present, dilated tubules (DTs) were retrieved; otherwise, slightly dilated tubules (SDT) (× 24) than that of the surroundings wereremoved. If no DT or SDT were found, not dilated tubules (NDTs) were excised. Testicular tissues were taken in a Petri dish 1 ml HEPES-buffered sperm medium (Ham’s F10 medium, Gibco BRL, Grand Island, NY, USA), and testicular biopsies were minced using sterile glass slides and shredded with 2 Jeweller forceps’s under an Olympus stereo microscope (SZ-PT, Tokyo, Japan) to separate individual tubules and then examined immediately under an inverted microscope (Olympus IMT2) with Hoffman optics modulation (× 400) for the presence of testicular spermatozoa in the entire Petri dish. Finally, testicular samples were processed using the erythrocyte lysing buffer [16]. The patients were followed up to 1 month in the form of weekly visits to detect any potential postoperative complications.

### Statistical Methods

Data were coded and entered using the statistical package for the Social Sciences (SPSS) version 26 (IBM Corp., Armonk, NY, USA). Data was summarized using frequencies (number of cases) and relative frequencies (percentages) for categorical variables. Comparisons between groups were done using unpaired *t* test [17]. Furthermore, Pearson’s correlation coefficient was used to determine the correlation between total motile sperms count and seminiferous tubular diameter.

## Results

**Fig. 2:**
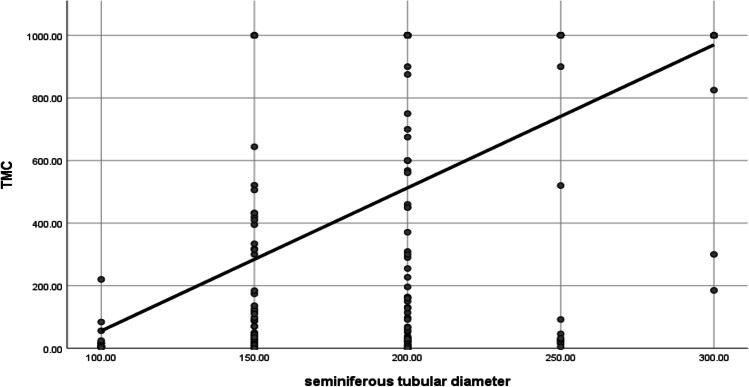
Correlation between seminiferous tubule diameter and total motile sperms count (TMSC)

The age and smoking status and hormonal profile and characteristics of the seminiferous tubular diameters and infertility history of the participants are detailed in Table 1. The participant’s testicular histopathology and testes volumes are listed in Table 2. The current study had demonstrated that 240 patients who underwent micro-TESE had eventful micro-TESE where they were naïve and salvage TESE. Dominating testicular histopathology in the group who had eventful micro-TESE were round spermatids arrest in 101 cases (42.1%) followed by SCO in 87 cases (36.3%) then primary spermatocytes in 33 cases (13.7%) and finally tubular hyalinization and atresia in 3 cases only (1.3%) (Table 2). The distribution of different seminiferous tubular diameters in different testicular histopathology is listed in Table 3. It should be noted that the highest mean TMSC was reported in the DTs group followed by the other study groups 941.72 ± 196.97, 487.37 ± 443.57, and 34.54 ± 60.79, respectively (Table 4). The change in the mean TMSC and different seminiferous tubular diameters was of high statistical significance (*p* < 0.001) (Table 4, Fig. 2).

**Table 1:** Age and smoking status and hormonal profile and characteristics of the seminiferous tubular diameters and infertility history of the participants

	Mean		Standard deviation	Median		Minimum		Maximum
Male age (years)	36.98		± 7.63	36.00		17.00		75.00
FSH mIU/ml	20.15		± 15.58	16.31		0.10		83.97
LH mIU/ml	10.40		± 7.56	7.74		0.01		48.54
Total testosterone ng/ml	3.77		± 1.92	3.48		0.23		12.76
Free testosterone ng/ml	24.27		± 14.37	21.99		1.10		125.00
Prolactin ng/ml	11.35		± 11.10	9.61		2.85		227.20
Estradiol pg/ml	30.26		± 15.96	29.00		2.60		184.00
Testosterone\E2 ratio	14.27		± 9.61	12.16		1.24		87.10
Right seminiferous tubule diameter	178.22		± 48.45	200.00		100.00		300.00
Left seminiferous tubule diameter	171.63		± 43.82	150.00		100.00		300.00
					Count		%	
Cigarette smoking		Yes			218		41.9%	
		No			302		58.1%	
Family history of infertility		Positive			11		2.1%	
		Negative			509		97.9%	
Previous assisted conception history		Positive			87		16.7%	
		No			433		83.3%	
Naïve/salvage micro-TESE		Salvage			278		53.5%	
		Naïve			242		46.5%	
Result of the current TESE		Positive			240		46.2%	
		Negative			280		53.8%

**Table 2:** Testicular histopathology of the participants and right and left testes volumes

				Count	%
Histopathology	Tubular hyalinization and atresia			10	2.0%
	Severe hypospermatogenesis			224	44.4%
	Severe hypospermatogenesis with the dominating histopathology as follow (*N* = 224)		Sertoli cell only	87	38.8%
			Round spermatids	101	45.1%
			Primary spermatocytes	33	14.7%
			Tubular hyalinization and atresia	3	1.4%
	Sertoli cell only			166	33.0%
	Mixed arrest			104	20.6%
	Mixed arrest (N = 104)		Sertoli cell only + round spermatid	2	0.4%
			Sertoli cell only + primary spermatocytes	18	3.5%
			Round spermatid	19	3.7%
			Primary spermatocytes + round spermatid	45	8.6%
			Primary spermatocytes	20	3.8%
Right testis volume		Normal (> 12.5 ml)		58	11.2%
		Small (< 12.5 ml)		449	86.3%
		Absent		13	2.5%
Left testis volume		Normal (> 12.5 ml)		59	11.3%
		Small (< 12.5 ml)		451	86.7%
		Absent		10	1.9%

**Table 3:** Frequency of different seminiferous tubular diameters in different testicular histopathology

Testicular histopathology		Different seminiferous tubular diameters		
		Non-dilated seminiferous tubules (NDT) cases (< 110 um)	Slightly dilated seminiferous tubules cases (SDT) (110–300 um)	Dilated seminiferous tubules cases (DT) (> 300 um)
Severe hypospermatogenesis	Round spermatid	0	85	16
	Sertoli cell only (SCO)	9	77	1
	Tubular hyalinization and atresia	2	1	
	Primary spermatocytes	3	24	6
Tubular hyalinization and atresia		5	5	0
Sertoli cell only		35	131	0
Round spermatid		0	17	2
Primary spermatocytes		0	20	0
Mixed pathology		6	58	1

**Table 4:** Correlation between different intraoperative seminiferous tubule diameters and total motile sperms count (TMSC)

		Non-dilated seminiferous tubules (NDT) cases (< 110 um)	Slightly dilated seminiferous tubules cases (SDT) (110–300 um)	Dilated seminiferous tubules cases (DT) (> 300 um)	*p* value
TMSC	Mean	34.54	487.37	941.72	< 0.001
	Standard deviation	± 60.79	± 443.57	± 196.97	
	Minimum	3.00	0.00	185.00	
	Maximum	220.00	1000.00	1000.00

**Fig. 3:**
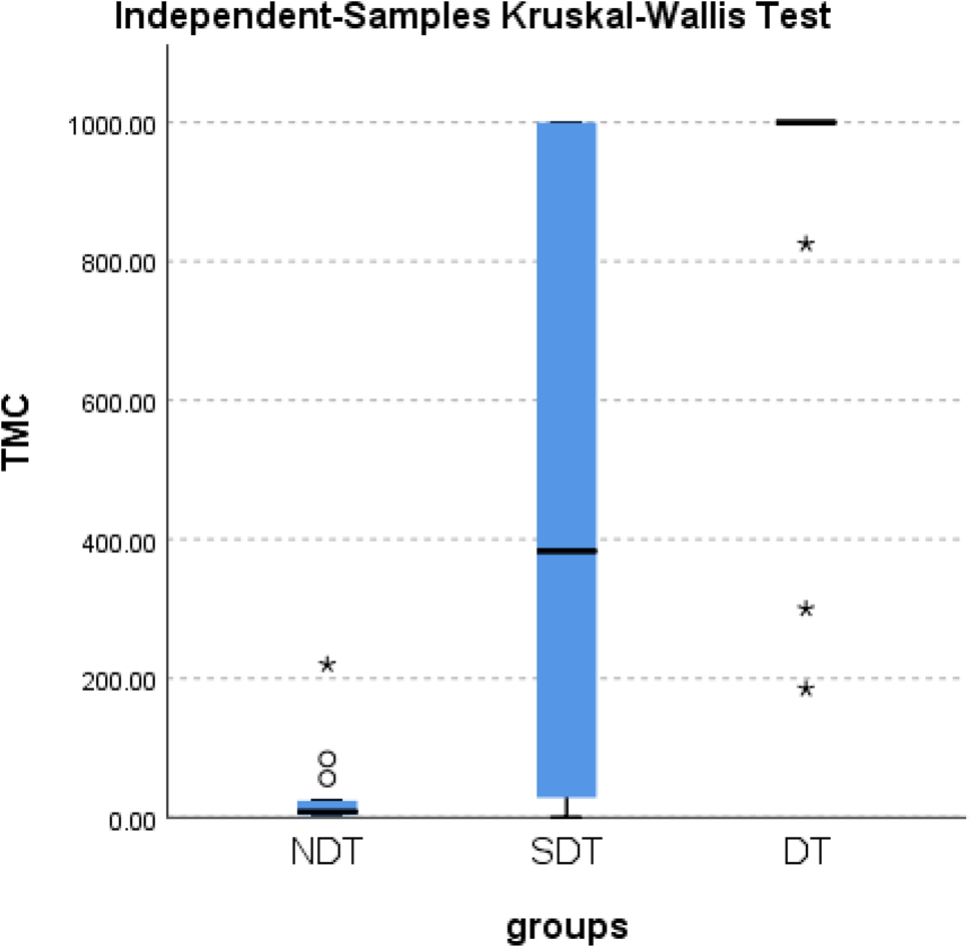
Intergroup correlations between different seminiferous tubular diameters and TMSC

Intergroup correlations between different seminiferous tubular diameters and TMSC were as follow: NDT versus SDT *p* < 0.001, NDT versus DT *p* < 0.001, and SDT versus DT *p* < 0.001, respectively (Fig. 3). Furthermore, 15 naïve cases had DTs and 12 (80.0%) of them had shown eventful micro-TESE. Conversely, 176 naïve cases had SDTs, and 91 (51.7%) of them had eventful micro-TESE. Only 8 (24.25%) cases of the 33 cases had NDTs and showed eventful micro-TESE (Table 5). The frequency of intrasurgical seminiferous tubular diameter and micro-TESE outcome among the fresh cases had demonstrated a highly statistical significance (*p* < 0.001) (Table 5). Interestingly, all salvaged cases (100%) with DTs and a previous eventful TESE had shown eventful TESE in the current study (Table 5). Also, the majority of salvaged cases (83.87%) with SDTs and a previous eventful TESE had shown eventful micro-TESE in the current study (Table 5). Only 3 (50%) cases out of 6 salvaged cases with NDTs and a previous eventful TESE had shown eventful micro-TESE in the current study (Table 5). The frequency of intrasurgical seminiferous tubular diameter and micro-TESE outcome among the salvaged cases with a previous eventful TESE had demonstrated a statistical significance (*p* 0.039) (Table 5).

**Table 5:** Correlation between sperm retrieval and different sizes of intrasurgical seminiferous tubular diameter in all cases

		Cases						*p* value
		Non-dilated seminiferous tubules (NDT) cases (< 110 um)		Slightly dilated seminiferous tubules cases (SDT) (110–300 um)		Dilated seminiferous tubules cases (DT) (> 300 um)		
		Count	%	Count	%	Count	%	
Naïve cases	Positive	8	24.25	91	51.7	12	80.0	< 0.001
	Negative	25	75.75	85	48.3	3	20.0	
Salvage cases with previous positive sperm retrieval	Positive	3	50	52	83.87	10	100	0.039
	Negative	3	50	10	16.13	0	0	
Salvage cases with previous negative sperm retrieval	Positive	3	14.3	46	25.56	1	100	0.149
	Negative	18	85.7	134	74.44	0	0	

## Discussion

The current study had demonstrated that 240 cases who were either naïve or salvage had eventful micro-TESE, and their histopathology was severe hypospermatogenesis that is defined by detecting an extremely low number of mature sperm cells to only immotile spermatozoa, detecting sperm cells in some (even in a single) of the testicular locations together with several hours of careful processing of the samples by experienced embryologists to retrieve sperm cells in the extracted testicular material. [18].

Noteworthy, the dominating histopathology in our cases with eventful micro-TESE was as follow round spermatids, SCO, primary spermatocytes, and finally tubular hyalinization and atresia. In the same context, Yu et al. (2018) reported similar findings as they stated that heterogenicity of histopathology is required for sperm retrieval [19]. Interestingly, our study had revealed that intraoperative DT (> 300 Um) is associated with eventful micro-TESE and the highest TMSC in cases with severe hypospermatogenesis. In the same context, 2 retrospective studies had shown similar findings. The first one was conducted by Caroppo et al. (2019) that was performed on naïve NOA patients who underwent micro-TESE and found that the pattern of seminiferous tubules together with testis histology predicted sperm retrieval [20]. Notably, they used that same magnification power (× 24) to evaluate the seminiferous tubules like ours in the current study [20]. Additionally, Caroppo et al. (2021) conducted another retrospective study on 79 salvage NOA patients who underwent micro-TESE using a higher magnification power (× 36) to evaluate the seminiferous tubules [21]. They stated similar findings delivered out by their previous study [20, 21]. Even though, not all fresh cases with DT in our study had shown eventful micro-TESE as highly dilated tubules (> 400 um) may sometimes be seen in testes of NOA patients showing impaired spermatogenesis with thickened lamina propria because of the increased extra cellular matrix. This could be seen in alignment with the morphometric study conducted by Volkmann et al. (2011) [22]. Consistently, it is worth mentioning that the apparent seminiferous tubules caliber may be misleading in patients with maturation arrest due to it’s homogenous appearance making it difficult for the surgeon to discriminate dilated seminiferous tubules compared to the surroundings. [21].

Furthermore, a recent Japanese study had shown that preoperative ultrasound determination of seminiferous tubular diameter is the strongest predictive factor for eventful micro-TESE [23]. However, the aforementioned study had shown that SRR in SCO was 10.7% which is lower than our finding (31.9%) as sperms were retrieved in 87 cases out of 273 cases (severe hypospermatogenesis and mixed arrest and SCO cases) where the majority of these cases were SDT[23]. Noteworthy, our study had shown that the SRR in SCO was 31.9% which is comparable to that reported by previous studies that were between 22.5 and 41% [1, 2, 24, 25]. In contrast, this finding could be seen lower than that delivered out by Amer et al. (2018) which was 49.8% [26]. However, it should be noted that the target population in the aforementioned study was threefold higher than the target population in the current study [26]. It should be mentioned that the current study had delivered out the following SRRs: 60.5% round spermatid cases, 31.9% SCO cases, 28.4% primary spermatocytes cases, and 23.1% tubular hyalinization and atresia cases, respectively. Thus, it had asserted that intraoperative seminiferous tubular diameter can ensure sperm retrieval with the highest TMSC especially in salvage cases. Additionally, SCO cases can be salvaged from repeated micro-TESE by using seminiferous tubular diameter as a predictive factor for sperm retrieval and to ensure the highest TMSC. So, patients will be saved from the potential complications such as temporary decrease in serum testosterone levels following micro-TESE that may warrant subsequent androgen replacement in 5–10% of these cases [4, 27]. In addition, hypo-echoic changes as early testicular ultrasound findings following micro-TESE while focal echogenic lesions of fibrosis and calcification as late findings at 6 months can be detected as expected complications [4]. Admittedly, the target population in the current study is not large that could be seen as major limitation of the current work.

However, the prospective nature of the current work adds strength to the current findings in contrast to the retrospective studies conducted by Caroppo et al. (2019) and (2021) [20, 21]. Also, the sample size was larger than the 2 aforementioned studies. [20, 21].

## Conclusion

Micro-TESE is still the gold standard surgical procedure together with the most dilated intra-operative seminiferous tubular diameter being essential for obtaining the highest TMSC in NOA patients including SCO cases.

## Data Availability

The data that support the findings of this study are not publicly available due to their containing information that could compromise the privacy of research participants. But, the data are available from the corresponding author S.F. (samehfayek@hotmail.com) upon reasonable request.

## Code availability

Data were coded and entered using the statistical package for the Social Sciences (SPSS) version 26 (IBM Corp., Armonk, NY, USA).

## References

[1] Caroppo E, Colpi EM, Gazzano G, Vaccalluzzo L, Scroppo FI, D'Amato G, Colpi GM (2017). Testicular histology may predict the successful sperm retrieval in patients with non-obstructive azoospermia undergoing conventional TESE: a diagnostic accuracy study. J Assist Reprod Genet.

[2] Bernie AM, Shah K, Halpern JA (2015). Outcomes of microdissection testicular sperm extraction in men with non-obstructive azoospermia due to maturation arrest. FertilSteril.

[3] Schlegel PN (1999). Testicular sperm extraction: micro-dissection improves sperm yield with minimal tissue excision. Hum Reprod.

[4] Amer M, Ateyah A, Hany R, Zohdy W (2000). Prospective comparative study between microsurgical and conventional testicular sperm extraction in non-obstructive azoospermia: follow-up by serial ultrasound examinations. Hum Reprod.

[5] Okada H, Dobashi M, Yamazaki T, Hara I, Fujisawa M, Arakawa S (2002). Conventional versus micro-dissection testicular sperm extraction for non-obstructive azoospermia. J Urol.

[6] Tsujimura A, Matsumiya K, Miyagawa Y, Tohda A, Miura H, Nishimura K (2002). Conventional multiple or microdissection testicular sperm extraction: a comparative study. Hum Reprod.

[7] Amer M, Zohdy W, Abd El Naser T, Hosny H, Arafa M, Fakhry E (2008). Single tubule biopsy: a new objective microsurgical advancement for testicular sperm retrieval in patients with non-obstructive azoospermia. Fertil Steril..

[8] Kamal A, Fahmy I, Mansour RT, Abou-Setta AM, Serour GI, Aboulghar MA (2004). Selection of individual testicular tubules from biopsied testicular tissue with a stereomicroscope improves sperm retrieval rate. J Androl.

[9] World Medical Association (2013). World Medical Association Declaration of Helsinki: ethical principles for medical research involving human subjects. JAMA.

[10] Schlegel PN, Su LM (1997). Physiological consequences of testicular sperm extraction. Hum Reprod.

[11] Ramasamy R, Schlegel PN (2007). Micro-dissection testicular sperm extraction: effect of prior biopsy on success of sperm retrieval. J Urol.

[12] Lambert B (1951). The frequency of mumps and of mumps orchitis. Acta Genet Stat Med.

[13] Sakamoto H, Saito K, Oohta M, Inoue K, Ogawa Y, Yoshida H (2007). Testicular volume measurement: comparison of ultrasonography, orchidometry, and water displacement. Urology.

[14] Sakamoto H, Saito K, Ogawa Y, Yoshida H (2007). Testicular volume measurements using Prader orchidometer versus ultrasonography in patients with infertility. Urology.

[15] Simoni M, Bakker E, Eurlings MC, Matthijs G, Moro E, Muller CR, Vogt PH (1999). Laboratory guidelines for molecular diagnosis of Y-chromosomal microdeletions. Int J Androl.

[16] Nagy ZP, Verheyen G, Tournaye H, Devroey P, Van Steirteghem AC (1997). An improved treatment procedure for testicular biopsy specimens offers more efficient sperm recovery: case series. FertilSteril.

[17] Chan YH (2003). Biostatistics102: Quantitative data – parametric & non-parametric tests. Singapore Med J.

[18] Hauser R, Yogev L, Amit A, Yavetz H, Botchan A, Azem F, Lessing JB, Ben-Yosef D (2005). Severe hypospermatogenesis in cases of non-obstructive azoospermia: should we use fresh or frozen testicular spermatozoa?. J Androl.

[19] Yu Y, Xi Q, Wang R, Zhang H, Li L, Liu R, Pan Y (2018). Heterogenicity of testicular histopathology and tubules as a predictor of successful microdissection testicular sperm extraction in men with non-obstructive azoospermia. Medicine (Baltimore)..

[20] Caroppo E, Colpi EM, Gazzano G (2019). The seminiferous tubule caliber pattern as evaluated at high magnification during microdissection testicular sperm extraction predicts sperm retrieval in patients with non-obstructive azoospermia. Andrology.

[21] Caroppo E, Castiglioni F, Campagna C (2021). Intrasurgical parameters associated with successful sperm retrieval in patients with non-obstructive azoospermia undergoing salvage microdissection testicular sperm extraction. Andrology.

[22] Volkmann J, Müller D, Feuerstacke C, Kliesch S, Bergmann M, Mühlfeld C, Middendorff R (2011). Disturbed spermatogenesis associated with thickened lamina propria of seminiferous tubules is not caused by dedifferentiation of myofibroblasts. Hum Reprod.

[23] Nariyoshi S, Nakano K, Sukegawa G, Sho T, Tsuji Y (2020). Ultrasonographically determined size of seminiferous tubules predicts sperm retrieval by microdissection testicular sperm extraction in men with non-obstructive azoospermia. Fertil Steril.

[24] Colpi GM, Colpi EM, Piediferro G (2009). Microsurgical TESE versus conventional TESE for ICSI in non-obstructive azoospermia: a randomized controlled study. Reprod Biomed Online.

[25] Deruyver Y, Vanderschueren D, Van der Aa F (2014). Outcome of micro-dissection TESE compared with conventional TESE in non-obstructive azoospermia: a systematic review. Andrology.

[26] Amer MK, Ahmed AR, Abdel Hamid AA, GamalEl Din SF (2019). Can spermatozoa be retrieved in non- obstructive azoospermic patients with high FSH level?: A retrospective cohort study. Andrologia.

[27] Ramasamy R, Yagan N, Schlegel PN (2005). Structural and functional changes to the testis after conventional versus microdissection testicular sperm extraction. Urology.

